# Laparoscopic repair of vesicovaginal fistula caused by radiation therapy: Use of a perirectal fat interposition graft

**DOI:** 10.1002/iju5.12284

**Published:** 2021-03-31

**Authors:** Tokumasa Hayashi, Auran Rosanne Cortes, Yugo Sawada, Shino Tokiwa, Mika Nagae, Nao Muta, Myat Noe Swe, Keo Nariroth, Masayoshi Nomura

**Affiliations:** ^1^ Department of Urogynecology Kameda Medical Center Kamogawa Chiba Japan

**Keywords:** interposition, laparoscopic repair, perirectal fatty tissue, vesicovaginal fistula

## Abstract

**Introduction:**

We present a case of laparoscopic repair of vesicovaginal fistula caused by radiation therapy using a perirectal fatty tissue interposition graft.

**Case presentation:**

A 72‐year‐old woman was diagnosed with vesicovaginal fistula induced by radiation therapy. Repair of the vesicovaginal fistula was achieved via laparoscopic approach. The fistula was exposed, followed by excision of fistula tract, fine dissection to achieve a traction‐free approximation of bladder mucosa, then water‐tight closure. An interposition graft derived from the perirectal fat was inserted to reduce the risk of repair failure. The patient did not have the incontinence problem at 1‐year follow‐up.

**Conclusion:**

The laparoscopic approach for vesicovaginal fistula repair is minimally invasiveness. Preparation of the interposition graft derived from the perirectal fatty tissue was easy and its mobility to achieve closure of the fistula was acceptable. Thus, this procedure is feasible for the repair of poorly vascularized tissues such as radiation‐induced fistulas.

**Abbreviations & Acronyms**
VVF: vesicovaginal fistula

**Keynote message**

VVF is the most devastating complication in the urogynecological setting. In particular, closure of radiation‐induced fistulas is difficult due to poor tissue quality. Interposition grafting of the perirectal fatty tissue is a feasible procedure in such cases.

## Introduction

VVF is a rare condition; however, is the most dreadful complication in the urogynecological setting. VVF usually occurs after hysterectomy or as a late complication of radiation therapy for uterine cancer.^1^ The success rate of the treatment for radiation‐induced VVF is lower compared with VVF due to other causes.^2^, ^3^ Herein, we present a case of radiation‐induced VVF which resulted in favorable clinical outcome following laparoscopic repair using perirectal fat interposition graft.

## Case presentation

**Fig. 1:**
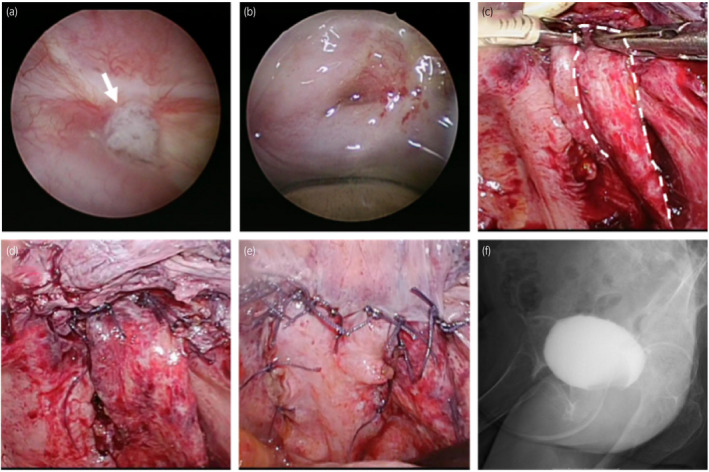
(a) Cystoscopic finding. The calculi (arrow) attached to the fistula. (b) The VVF was located at the vaginal cuff. (c) The graft was made using perirectal fatty tissue (encircled by dotted line). (d) The graft was interposed between the suture points of bladder and vagina. (e) Surrounding peritoneum was sutured. (f) No leakage was observed during cystography done after operation.

A 72‐year‐old woman presented to our clinic with a complaint of constant leakage of urine. At 37 years of age, the patient underwent radical hysterectomy and radiation therapy for uterine cervical cancer. On cystoscopic finding, the hollow area was observed at the inter‐ureteric ridge of the bladder near the right ureteral orifice, and there were bladder calculi attached (Fig. 1a). Also, the saline infused into the bladder during cystoscopy leaked into the vagina. Under the direct vision using speculum, the leakage was observed from the small hole at the vaginal cuff, with pale surrounding tissue (Fig. 1b). She was subsequently diagnosed with VVF as a late complication of radiation therapy.

Before the laparoscopic approach, a guidewire was placed through the fistula, and ureteral stents were inserted bilaterally. Bladder calculi were removed transurethrally. The patient was placed in the 25° Trendelenburg position and four trocars were used (on the umbilical top, halfway between the umbilicus and the pubic symphysis, and in the left and right iliac fossa). The bladder wall was opened transversely near the fistula. Next, the fistula tract was excised up to the healthy tissue margin, and was then finely dissected between the bladder and vaginal wall to obtain a tension‐free approximation of the bladder mucosa. The vaginal wall was closed by the one‐layer interrupted suturing technique using 3‐0 Vicryl sutures. Subsequently, water‐tight closure of the bladder wall was ensured by placing interrupted sutures using 4‐0 Vicryl with tension‐free approximation at the bladder wall closing point.

**Fig. 2:**
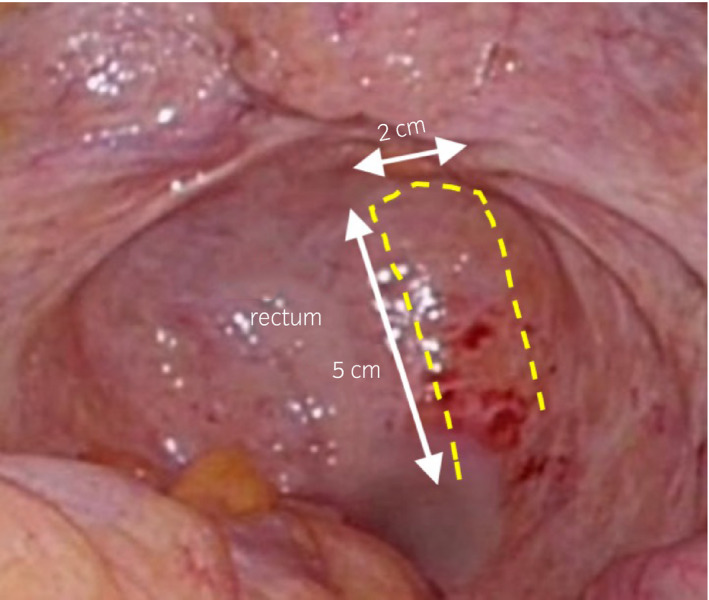
The illustration of perirectal fat interposition graft. The graft was made using perirectal fatty tissue. The peritoneum on the right side of the rectum was cut, until about 5 cm in length and 2 cm in width of the perirectal fat was freely mobilized.

Next, the graft was prepared for interpositioning between the bladder and the suture point in the vagina. The graft was made using perirectal fatty tissue. The peritoneum on the right side of the rectum was cut, until about 5 cm in length and 2 cm in width of the perirectal fat was freely mobilized. (Fig. 1c). Figure 2 is the illustration of the perirectal flap. After assessment of the mobility, the edge of the graft was fixed using 3‐0 Vicryl sutures between the suture points of the bladder and vagina (Fig. 1d). Finally, water‐tight closure was re‐assessed by infusing saline transurethrally, and the peritoneum surrounding the closure area was sutured and covered over (Fig. 1e). The operation finding is attached as Supporting Information (Video S1).

The surgical time was 5 h and 32 min (the pneumoperitoneum time was 4 h and 10 min), and blood loss was 10 mL. No perioperative complications were observed. On postoperative day 12, complete closure of the fistula was confirmed by cystography (Fig. 1f), and the urethral catheter was removed. No recurrence of incontinence was reported at 1‐year postoperative follow‐up.

## Discussion

The choice of the surgical approach, transvaginal or transabdominal, for the treatment of VVF depends on the ability and experience of the surgeon. The advantage of transvaginal approach lies in its un‐invasiveness, faster recovery, and the aspect of cosmetic issue compared with the laparotomy approach. In contrast, considering the small surgical field, surgeons with limited experience in the transvaginal approach may face difficulties during suturing.^4^ The transabdominal approach should be selected in cases of poor vaginal access, fistulas located high in the supratrigonal area, and/or proximity between the fistula and ureteral orifice.^5^

Advantage of laparoscopic repair includes low operative morbidity, less analgesic use, and shorter hospital stay. The surgical vision under laparoscopy was superior to that with the naked eye under open laparotomy, which enabled precise suturing. We chose the laparoscopic repair technique because the procedure could be performed in the same field of view as laparoscopic sacrocolpopexy, which has been performed on patients with pelvic organ prolapse in our department.^6^ Studies have reported a cure rate of 86–100% following laparoscopic VVF repair.^7^, ^8^, ^9^ However, there are no randomized controlled trials comparing laparoscopic and open laparotomy repair of VVF due to the limited number of cases of this condition.^10^

Laparoscopic repair of VVF is based on the classical laparotomy technique described by O’Conor, which involves bi‐valving the bladder with a subsequent transvesical approach.^11^ Subsequently, Rizvi *et al*. proposed the modified laparoscopic abdominal VVF repair, “Mini‐O’Conor” technique, wherein a limited cystotomy of <2 cm size is made close to the fistula tract.^12^ We followed this procedure, which has more advantage such as better view of operative field. Bladder pain and severe hematuria were not observed after the surgery. This could be attributed to the small cystotomy that could have potentially reduced bladder spasms.

To increase the success rate of closure of fistula, there is an opinion on utilization of interposition flaps. Interposition flaps have been hypothesized to function as a barrier, fill the dead space, establish vascularity to improve tissue growth and maturation, thus reinforcing the repair.^13^ A study reported on open laparotomy repairs of VVF in 37 women with and without interposition flaps. Continence was achieved in all cases where the interposition flap was used, as opposed to 64% of the women in whom the flap was not utilized.^13^ There are pros and cons of interposition flaps pertaining to laparoscopic VVF repair. Theoretically, these flaps establish vascularity to improve tissue growth; therefore, could be beneficial in cases associated with poor tissue quality, such as fistulas arising due to previous surgical failure or radiation therapy.

The omentum has been commonly utilized for interposition flaps; however, creating the flap in the 25° Trendelenburg position would not be easy because the omentum moves cephalad with the bowel. We prepared the interposition flap derived from the right perirectal fatty tissue. The vascularity and thickness for the flap were adequate and its mobility to achieve closure was acceptable. It is speculated that the arterial supply of these tissues is most likely from the superior rectal or middle rectal artery. During preparation of flap, careful dissection should be observed in order to avoid inadvertent damage to the hypogastric nerve, right ureter, and rectal wall. We believe that this procedure is feasible for the repair of poorly vascularized tissues such as radiation‐induced fistulas (e.g. VVF).

## Conflict of interest

The authors declare no conflict of interest.

## Supporting information


**Video S1.** Short video clip of laparoscopic repair for VVF using perirectal fatty tissue.

## References

[1] Mraz JP , Sutory M . An alternative surgical treatment of post‐irradiation and rectovaginalfistulas: the serp‐muscular interstitial graft (patch). J. Urol. 1994; 151: 357–9. 8283524 10.1016/s0022-5347(17)34948-0

[2] Pushkar DY , Dyakov VV , Kasyan GR . Management of radiation‐induced vesicovaginal fistula. Eur. Urol. 2009; 55: 131–8. 18486313 10.1016/j.eururo.2008.04.044

[3] Hilary JH , Osman NI , Hilton P *et al*. The aetiology, treatment, and outcome of urogenital fistulae managed in well ‐and low‐resourced countries: a systematic review. Eur. Urol. 2016; 70: 478–92. 26922407 10.1016/j.eururo.2016.02.015

[4] Singh V , Shiha RJ , Sankhwar SN *et al*. Transvaginal repair of complex and complicated vesicovaginal fistulae. Int. J. Gynecol. Obstet. 2001; 114: 51–5. 10.1016/j.ijgo.2011.01.015 21529805

[5] Breen M , Ingber M . Controversies in the management of vesicovaginal fistula. Best Pract. Res. Clin. Obstet. Gynaecol. 2019; 54: 61–72. 30037532 10.1016/j.bpobgyn.2018.06.005

[6] Moriyama S , Huang TW , Sittidilokratna K *et al*. Clinical outcomes of laparoscopic sacrocolpopexy for pelvic organ prolapse: a retrospective analysis of 500 cases from a single institution. Jpn. J. Urol. 2017; 108: 137–44. 10.5980/jpnjurol.108.137 30033976

[7] Sotelo R , Mariano MB , Garcia‐Segui A *et al*. Laparoscopic repair of vesicovaginal fistula. J. Urol 2005; 173: 1615–8. 15821510 10.1097/01.ju.0000154701.97539.ef

[8] Das Mahapatra P , Bhattacharyya P . Laparoscopic intraperitoneal repair of high‐up urinary bladder fistula: a review of 12 cases. Int. Urogynecol. J. 2007; 18: 759–62. 10.1007/s00192-006-0215-6 17031487

[9] Abdel‐Karim AM , Mousa A , Hasouna M , Elsalmy S . Laparoscopic transperitoneal extravesical repair of vesicovaginal fistula. Int. Urogynecol. J. 2011; 22: 693–7. 21107809 10.1007/s00192-010-1334-7

[10] Miklos JR , Moore RD , Chinthakanan O . Laparoscopic and robotic ‐assisted vesicovaginal fistula repair: a systematic review of the literature. J. Min. Inv. Gynecol. 2015; 22: 727–36. 10.1016/j.jmig.2015.03.001 25764976

[11] O’Conor VJ . Review of experience with vesicovaginal fistula repair. J. Urol. 1980; 123: 367–9. 7359640 10.1016/s0022-5347(17)55939-x

[12] Rizvi SJ , Gupta R , Patel S *et al*. Modified laparoscopic abdominal vesico‐vaginal fistula repair‐ “Mini‐O’Conor” vesicotomy. J. Laparoendosc. Adv. Surg. Tech. 2010; 20: 13–5. 10.1089/lap.2009.0176 20059325

[13] Evans DH , Madjar S , Politano VA *et al*. Interposition flaps in transabdominal vesicovaginal fistula repairs: are they really necessary? Urology 2001; 57: 670–4. 11306377 10.1016/s0090-4295(01)00933-5

